# Effect of Defatting and Extraction Solvent on the Antioxidant and Pancreatic Lipase Inhibitory Activities of Extracts from *Hermetia illucens* and *Tenebrio molitor*

**DOI:** 10.3390/insects12090789

**Published:** 2021-09-03

**Authors:** Joaquín Navarro del Hierro, Emma Cantero-Bahillo, Tiziana Fornari, Diana Martin

**Affiliations:** 1 Departamento de Producción y Caracterización de Nuevos Alimentos, Instituto de Investigación en Ciencias de la Alimentación (CIAL) (CSIC–UAM), 28049 Madrid, Spain; joaquin.navarrodel@uam.es (J.N.d.H.); emma.cantero@uam.es (E.C.-B.); tiziana.fornari@uam.es (T.F.); 2 Sección Departamental de Ciencias de la Alimentación, Facultad de Ciencias, Universidad Autónoma de Madrid, 28049 Madrid, Spain

**Keywords:** *Tenebrio molitor*, *Hermetia illucens*, edible insects, defatting, ultrasound-assisted extraction, antioxidant, pancreatic lipase, amino acids, sterols, lipids

## Abstract

**Simple Summary:**

The food industry is notably investing more resources on the production of nutritious, healthy, safe and sustainable products derived from edible insects. In this sense, natural extracts (or concentrated forms of compounds from natural sources) are usually food ingredients with added value for human health. This is due to their intrinsic beneficious biological activities; however, bioactive extracts from edible insects have been scarcely explored. Due to that and considering that the bioactivities of extracts might be conditioned by parameters of the technological process, we assessed how different extraction conditions, such as the defatting of the raw insect flours or the extraction solvents employed, affected two bioactivities of the resulting extracts from insects: the blocking of the digestion of fats from the diet by evaluating the inhibition of the responsible enzyme (pancreatic lipase), as well as their antioxidant activity. *T. molitor* and *H. illucens* were used, as they are two of the most known edible species for both feed and food. We observed a multibioactivity for all the extracts. Both tested processing factors differentially modulated the bioactivity of extracts from both species. We also analysed the composition of the *H. illucens* extracts and detected amino acids, lipids, carbohydrates, sterols and organic acids.

**Abstract:**

The production of specific insect extracts with bioactive properties for human health is an emerging and innovative field for the edible insects industry, but there are unexplored extraction factors that might modulate the bioactivity of the extracts. Ultrasound-assisted extracts from *T. molitor* and *H. illucens* were produced. Effects of defatting pre-treatment and extraction solvent were evaluated on extraction yield, antioxidant activity and pancreatic lipase inhibitory effect. Chemical characterisation of defatted extracts from *H. illucens* was performed by GC-MS-FID. Non-defatted extracts showed higher extraction yields. Defatted extracts had similar extraction yields (around 3%). Defatted extracts had higher antioxidant activity, *T. molitor* being stronger than *H. illucens*. Antioxidant activity of *T. molitor* methanol extract was higher than the rest of solvents. Aqueous ethanol improved the antioxidant activity of *H. illucens* extracts. All extracts inhibited lipase, but no significant effect of defatting and solvent was observed for *T. molitor*. A significant higher inhibitory activity was observed for *H. illucens*, the strongest being defatted 100% and 70% ethanol *H. illucens* extracts. *H. illucens* extracts contained free amino acids and disaccharides, together with minor fractions of lipids, sterols and organic acids. These results evidence the potential of extracts obtained from edible insects as antioxidants and inhibitors of the pancreatic lipase, a simultaneous multibioactivity that might be favoured by the defatting pre-treatment of the samples and the solvent of extraction.

## 1. Introduction

Current popularity towards edible insects has been related to their role as an alternative to traditionally consumed fats and proteins from animal sources, as they cover the dietary needs of humans with the additional advantage of being a sustainable food with a low environmental impact [1]. Due to that, the European Food Safety Authority (EFSA) considered for the first time ‘whole insects and their parts’ as novel foods from 1 January 2018 based on the new Regulation (EU) 2015/2283 [2]. EFSA suggested a list of edible insects with a strong potential to be used as food and feed in the European Union, such as *Musca domestica, Acheta domesticus, Tenebrio molitor, Hermetia illucens, Bombyx mori, Gryllodes sigillatus, Achroia grisella, Alphitobius diaperinus, Galleria mellonella, Schistocerca Americana, Locusta migratoria migratorioides* and *Zophobas atratus* [3]. As a relevant milestone, in January 2021 EFSA ruled for the first time on the first application for a novel food made from insects, specifically *T. molitor*, concluding a positive opinion considering that it is safe under the proposed uses and use levels [4]. At the moment of writing the current manuscript (June 2021), the Standing Committee on Plants, Animals, Food and Feed (Novel Food and Toxicological Safety section), from the European Commission has authorised the production of dried yellow mealworm as a novel food [5].

Together with their nutritional value, evidence show that edible insects contain a huge diversity of other micronutrients and compounds different from fats and proteins, such as fibres, minor lipid compounds, phenolic compounds, alkaloids and other compounds yet to be discovered. In fact, multiple different bioactivities are being reported recently in the literature for edible insects, including anti-inflammatory, antiproliferative, antioxidant, antiangiogenic or antimicrobial, although studies are still scarce [6,7,8,9,10,11,12,13].

Taking into account these evidence, the production of specific insect extracts would be a way to produce concentrated forms rich in bioactive compounds, which might be worth to study in order to offer innovative insect products for human health and to potentiate the development of insect-based food products. In this sense, and following the trend in the research field of natural extracts typically obtained from plants, non-conventional extraction processes might be proposed as green techniques to produce extracts derived from insects of current interest. These techniques require reduced energy consumption, extraction times and have an increased extraction efficiency [14], and for these reasons, they contribute to the sustainability principle of the industry of edible insects. As an example, we have recently produced different non-defatted extracts from *A. domesticus* and *T. molitor* by ultrasound-assisted extraction or pressurised liquid extraction, and also evaluated the effect of different green solvents such as ethanol or aqueous ethanol on their antioxidant and pancreatic lipase-inhibitory activity [15,16]. We effectively demonstrated that the antioxidant activity of these extracts was similar for both species regardless of the method of extraction, even though solvents of increased polarity such as aqueous ethanol were preferred for a higher antioxidant activity. On the other hand, we also evidenced for the first time that extracts from these edible insects were able to inhibit the pancreatic lipase, which is a useful mechanism to limit the absorption of dietary lipids. This enzyme-inhibitory activity had never been reported before for edible insects nor enriched products presented as insect extracts. We were able to conclude that extracts from the insect *T. molitor* seemed to be more effective than *A. domesticus* [16]. These evidence show that different extraction conditions and other species of edible insects are worth to be further explored in order to obtain multibioactive insect extracts.

Concerning the production of natural extracts, it should be considered that when the raw sources are rich in other macronutrients, such as proteins, carbohydrates or lipids, the minor fraction responsible for the bioactive effects might suffer a ‘diluting effect’ and its potential bioactivity be masked, especially when these macronutrients are co-extracted during the process. In the specific case of insects, despite they are known for having a high protein content, their additional richness in lipids is also a relevant component to consider when consumed under their larval form, as occurs for *T. molitor* and *H. illucens* [17]. Therefore, during the production of bioactive extracts from these species, a high co-extraction of either lipids or proteins might take place, an outcome that will be conditioned by the solvent and conditions of extraction. For this reason, the evaluation of the impact of defatting the insect flour on the subsequent bioactivity of the resulting extracts would be of interest in order to move one step forward in the development of efficient extracts from insects. Additionally, this would also be of interest given that the defatting process of insect flours is a relevant issue of this industry, since the main current production of insect-based products for feed and food is aimed at obtaining protein-rich products, and a previous defatting step is performed. Therefore, the exploration of the defatting process would have the double interest of obtaining defatted raw material to produce protein-rich products as well as concentrated extracts in other minor compounds of interest. However, the impact of such defatting effect on the subsequent bioactivity of the products has not been tested by specific comparative studies with the equivalent non-defatted samples, since it has been generally assumed that a previous defatting process is necessary for the production of insect flours with bioactivity, as antioxidant or antimicrobial activities [18,19].

Concerning other species of edible insects, *H. illucens* is, together with *T. molitor* and *A. domesticus*, one of the most popular edible insects currently produced and investigated for both animal feed and human food. In addition, *H. illucens* is being used as a bioconversion tool for waste management [20,21,22,23]. However, previous studies focusing on concentrated extracts obtained from *H. illucens* by using advanced methods of extraction aimed at producing potential bioactive ingredients for human health have not been described. This would be of great interest, considering that, similar to other edible insects, *H. illucens* has also been suggested as a species with bioactive properties [24,25,26].

The aim of this work was to evidence the impact that the process of defatting prior to extraction, as well as the solvent of extraction, might have on the production of bioactive extracts from edible insects in their larva stage. Different isolated experiments testing the variable factors were performed. First, the insect *T. molitor* was used to show the effect of defatting of the raw material on the subsequent bioactivity of extracts obtained with three solvents of different polarity: methanol, ethanol and ethyl acetate. Then, under fixed conditions of ethanol extraction, a comparative study between extracts of *T. molitor* and *H. illucens* was performed in order to evaluate the ‘defatting’ and ‘species’ factor on the resulting bioactivities. Finally, defatted *H. illucens* was selected to delve deeper into the effect of using variable proportions of ethanol as solvent of extraction. For all the assays, ultrasound-assisted extraction was used as the method of extraction. The antioxidant activity and inhibitory effect on pancreatic lipase of the resulting extracts were evaluated. Additionally, the defatted extracts obtained from *H. illucens* were selected to perform their characterisation by GC-MS-FID.

## 2. Materials and Methods

### 2.1. Raw Materials and Chemicals

Dry larvae of *T. molitor* and *H. illucens* were purchased from online distributors (Highridge and Chubby Mealworms, respectively). Prior to extraction, insects were ground in a knife mill (Grindomix GM 200, Retsch GmbH, Haan, Germany) and stored at room temperature in sealed bags protected from light, oxygen and moisture until further use.

Absolute ethanol was from Panreac (Barcelona, Spain). Hexane (95%), methanol and ethyl acetate were acquired from Macron (Gliwice, Poland). N,O-bis-(trimethylsilyl)trifluoroacetamide (BSTFA), 2,2-diphenyl-1-picrylhydrazyl (DPPH), Dulbecco’s phosphate buffered saline (PBS), 4-methylumbelliferyl oleate (4-MUO) and lipase from porcine pancreas were from Merck KGaA (Darmstadt, Germany).

### 2.2. Defatting of Samples

A conventional solid-liquid extraction was performed with 95% hexane at a ratio of sample to solvent of 1:5 (*w*/*v*) in an Ultraturrax (11,000 rpm) for 5 min. Then, the mixture was centrifuged at 4500 rpm for 10 min at 20 °C. The supernatant was removed, and the precipitate was extracted again following the same procedure. The final precipitate sample was completely dried to remove any residual hexane by flushing with nitrogen gas for 5 min and heating at 30 °C for 10 min in an orbital incubator.

### 2.3. Production of the Extracts

Both defatted and non-defatted samples from both insect species were extracted employing direct sonication (Branson SFX250 Digital Sonifier, Branson Ultrasonics, Danbury, CT, USA) at an amplitude of 60% (sonication output) in continuous pulse at 20 kHz with an ultrasonic probe (1/2″ diameter), as described by Navarro del Hierro et al. [16]. In the first experiment, samples from *T. molitor* were extracted with either methanol, ethanol or ethyl acetate. These solvents were chosen as typical solvents of different polarities used in the general field of natural extracts. In the second experiment, samples from *H. illucens* were extracted with ethanol for comparative purposes with the ethanol extracts from *T. molitor*. Additionally, in the third experiment, samples from *H. illucens* were also extracted with a 70% ethanol solution and 50% ethanol solution in order to delve deeper into the effect of using variable proportions of ethanol as solvent of extraction. In all cases, extractions were performed at a ratio of sample to solvent of 1:10 (*w*/*v*) for 15 min. The temperature during the extraction process was kept under 70 °C. The mixture was submitted to centrifugation at 3400× *g* for 10 min. The organic solvent in the supernatant was rotary evaporated, while the aqueous fraction of the ethanol:water extracts was further lyophilised. Extraction yield (%, *n* = 2) was estimated as the weight of crude dried extract obtained with respect to the weight of ground insect used for extraction and final dried extracts were kept at −20 °C until further use.

### 2.4. Antioxidant Activity of the Extracts by DPPH Assay

The antioxidant activity of the extracts was measured by the DPPH˙ assay [27]. A solution of DPPH˙ in methanol (0.06 mM) was prepared and 560 μL of this solution was mixed with 40 μL of each of the extracts in methanol at 5 mg/mL. Reaction mixture was vortexed and left in the dark at RT for 60 min. Absorbance of the mixture was measured at 517 nm and methanol was used as control. Control samples were made in the absence of extracts by employing the same steps. The concentration of remaining DPPH˙ in the samples was determined from a DPPH˙ calibration curve. Experiment was repeated two times for each extract. The antioxidant activity was calculated as the percentage of inhibition of DPPH˙ radical employing the following formula:

% Inhibition DPPH = 100 − [(μg DPPH·/mL _sample_/μg DPPH·/mL _control_) × 100]

### 2.5. Pancreatic Lipase Inhibition Assay

The inhibitory activity of pancreatic lipase was measured according to Herrera et al. [28] with modifications. A solution of each of the extracts at 5 mg/mL was prepared. Reaction mixture consisted of 500 μL of extract solution in PBS/DMSO (1.7:1 *v*/*v*) at 5 mg/mL, 500 μL of freshly prepared pancreatic lipase at 1 mg/mL (10 mg of lipase in 10 mL of PBS, stirred for 10 min and centrifuged at 4000 rpm for 10 min) and 1 mL of 4-MUO solution at 0.1 mM in PBS. Extracts were at a final concentration of 1.25 mg/mL in the reaction medium. Control samples were prepared following the same procedure but in absence of extracts. Each sample was prepared in triplicate.

An orbital incubator was used to incubate the samples at 250 rpm and 37 °C for 20 min. After, three aliquots of 150 μL were added to a 96-well plate. A fluorescence microplate reader (Polarstar Galaxy, BMG Labtechnologies, Offenburg, Germany) was used to measure the amount of hydrolysed 4-MUO by lipase, setting an excitation wavelength of 350 ± 10 nm and an emission wavelength of 450 nm. The inhibition of pancreatic lipase activity was determined as follows:

% Inhibition Lipase = 100 − [(Fluorescence_sample_/Fluorescence_control_) × 100]

### 2.6. Analysis of the H. illucens Extracts by GC-MS

Taking into account the results obtained for all the extracts in terms of their biological activities, the ethanol and aqueous ethanol extracts of *H. illucens* were selected for a deeper evaluation on their composition by GC-MS. Extracts from *H. illucens* were previously derivatised with BSTFA according to Herrera et al. [29] with small modifications. Samples were solubilised at 10 mg/mL in BSTFA and heated for 1 h at 75 °C. Then, samples were allowed to settle for 5 min at room temperature, followed by centrifugation for 5 min at 4500 rpm (Centrifuge MiniSpin^®^ plus, Eppendorf AG, Hamburg, Germany). The isolated supernatant was analysed in an Agilent 7890A GC-MS (Agilent Technologies, Santa Clara, CA, USA). An Agilent HP-5MS UI capillary column (30 m × 0.250 mm × 0.25 μm) was used, and helium was employed as the carrier gas at a flow of 2 mL/minute. Total of 1 μL of derivatised sample was injected in splitless in a G4513A autoinjector, setting the inlet temperature at 260 °C. The temperature program was as follows: 50 °C, increased at 10 °C/minute to 310 °C and finally held for 25 min. The temperatures at the MS ion source and the interface were 230 °C and 280 °C, respectively. A mass range of 30–1000 amu and a scanning speed of 0.79 scans/s were used. Compounds were identified by the NIST MS Data library, by the mass spectra according to literature, or according to commercial standards, which were also derivatised employing the same steps as done with the samples. Quantification of compounds was performed by calibration curves obtained from commercial standards whenever possible. Oleic acid was used for the quantification of fatty acids, 1-oleoyl-rac-glycerol was used for monoacylglycerides, lysine was used for amino acids, sucrose was used for disaccharides, myo-inositol was used for inositol, isocitric acid was used for organic acids and β-sitosterol was used for sterols.

### 2.7. Statistical Analysis

Statistical analyses were performed by means of the general linear model procedure of the SPSS 26.0 statistical package (SPSS Inc., Chicago, IL, USA) by one-way analysis of variance. Differences were considered significant at *p* ≤ 0.05. Post-hoc Tukey’s tests were performed in order to establish significant differences. Pearson correlation tests were conducted for additional analyses.

## 3. Results & Discussion

### 3.1. Effect of Defatting and Solvent of Extraction on the Yield and Bioactivity of Extracts of T. molitor

In the first experiment, the insect *T. molitor* was tested to show the effect of defatting process and solvent of extraction on the subsequent bioactivity of the extracts. A conventional defatting process with hexane was performed for the defatted samples, followed by ultrasound-assisted extraction (UAE).

**Figure 1:**
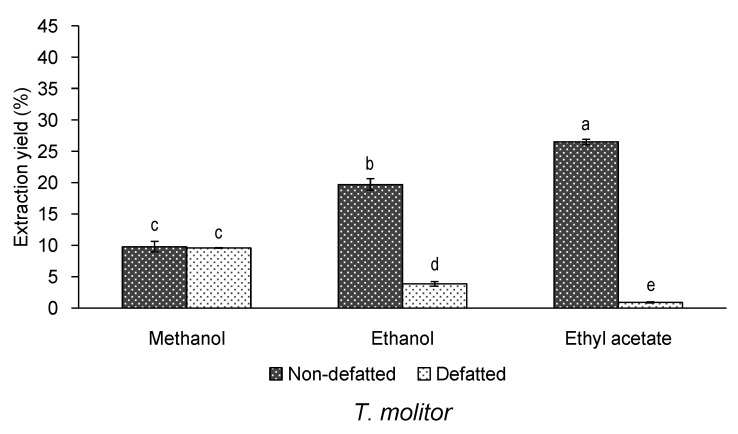
Extraction yield (%, *n* = 2) of non-defatted or defatted *T. molitor* extracts obtained by ultrasound-assisted extraction with different solvents of extraction. Standard deviations are indicated by error bars. Mean values with different letters are significantly different (*p* ≤ 0.05).

First, taking into account the relevance of the extraction yield in the field of natural extracts, this parameter was evaluated (Figure 1). As expected, the defatting process had a significant effect on the extraction yield of *T. molitor* (*p* = 0.003). Thus, as indicated in Figure 1, non-defatted samples showed, in general, higher extraction yields than defatted samples. This result was expected, since the used extraction solvents have a low to moderate polarity, hence they might favour the extraction of lipid compounds. Thus, in the case of non-defatted samples of *T. molitor*, the highest extraction yield was obtained with the less polar solvent, namely ethyl acetate (around 27%). On the contrary, for defatted samples, ethyl acetate was the least efficient for producing extracts, due to the lack of residual non-polar compounds available to be extracted. Due to that, in the case of defatted samples, the extraction yield increased along with the polarity of the solvent, methanol being the one that guaranteed the maximum extraction yield (closer to 10%).

**Figure 2:**
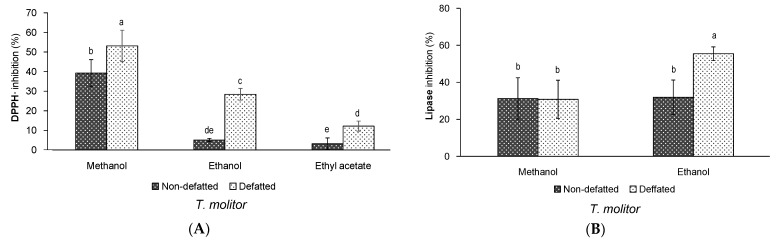
(**A**) DPPH inhibition (%, *n* = 6) and (**B**) pancreatic lipase inhibition (%, *n* = 3) of non-defatted or defatted *T. molitor* extracts obtained by ultrasound-assisted extraction with different solvents of extraction. Standard deviations are indicated by error bars. Mean values with different letters are significantly different (*p* ≤ 0.05).

The effect of defatting process and solvent of extraction on the antioxidant activity of the extracts from *T. molitor* is shown in Figure 2A. All the extracts had antioxidant activity, but the magnitude of such activity was different depending on the variability factors. Thus, a significant effect of the defatting process was detected regardless of the extraction solvent (*p* = 0.014), showing that the antioxidant activity of the defatted extracts was higher than that from non-defatted ones. A significant effect of the solvent of extraction was also observed (*p* < 0.001). Thus, regardless of the defatting process, the antioxidant activity of those extracts obtained with methanol was higher than the extracts obtained with the other two solvents.

Therefore, considering all the factors together, the highest antioxidant activity of *T. molitor* extracts was achieved when the samples were initially defatted and subsequently extracted with methanol. Nevertheless, it is interesting to remark that the inhibitory values of both methanol extracts was not exceedingly different (around 40% inhibition for non-defatted extracts and around 50% for defatted extracts) (Figure 2A). This would suggest the possibility of producing interesting extracts from *T. molitor* in terms of their antioxidant effects without performing an additional defatting step, at least when methanol is used as the solvent of extraction. However, if the production of antioxidant extracts is sought by using greener extraction solvents, as ethanol might be, then a previous defatting step might be worth to be performed, since the antioxidant activity would be increased from around 5% to 30%, approximately.

Previous studies showing the effect of defatting of flours from insect extracts on the antioxidant activity have not been described, since the production of insect extracts is quite novel and scarcely explored. The obtained results in the current study suggest that the main compounds responsible for the antioxidant activity of *T. molitor* might be mainly of high or moderate polarity, since the removal of lipids seems, in general, to improve the antioxidant activity of insect products. In a previous study, we already found that other different insect extracts obtained from non-defatted flours with more polar solvents (aqueous ethanol) were more efficient in terms of their antioxidant activity compared to ethanol [16]. However, in such study we did not address the comparison with defatted samples nor a wide range of solvents. On the other hand, different studies have evidenced the antioxidant activity of defatted insect flours [18,19]. Unfortunately, these studies did not compare such effect with non-defatted samples, since it has been generally assumed that a previous defatting process is necessary for the obtention of insect flours with antioxidant activity. In this sense, our study shows that it is possible to obtain non-protein antioxidant extracts from non-defatted and defatted insect flours with variable, and even similar, antioxidant capacities depending on the solvent of extraction. Further studies would be of interest to clearly characterise these extracts and elucidate the main compounds responsible for the antioxidant effects. In this sense, in our previous study we found a positive correlation between the antioxidant activity of the insect non-defatted extracts and their value of total phenolic compounds [16].

The ability of *T. molitor* extracts to inhibit the pancreatic lipase enzyme is shown in Figure 2B. Taking into account the low extraction yield and poor antioxidant activity of the extracts obtained with ethyl acetate, only those extracts produced with methanol and ethanol were tested for this bioactivity. According to Figure 2B, all the extracts showed the ability to inhibit the pancreatic lipase, although as opposed to the antioxidant activity, a lack of significant effect of the defatting process and the extraction solvent was observed (*p* > 0.05 for both factors). Therefore, this would suggest that non-defatted and defatted *T. molitor* extracts are able of inhibiting the pancreatic lipase in a similar way, regardless of the extraction solvent used during UAE. Nevertheless, when considering all the variability factors together, the defatted extract obtained with ethanol, which exhibited a lipase inhibition mean value of around 56%, stood out compared to the rest of extracts (Figure 2B). The obtained inhibition values could be considered of great potential, as they are similar to other natural extracts from plant sources, which have been typically explored for this bioactivity [30].

The inhibition of the pancreatic lipase by edible insects is a quite novel bioactivity that we reported for the first time for non-defatted extracts of *T. molitor* and *A. domesticus* [16]. In such study, we demonstrated that the extracts from *T. molitor* were more effective than those from *A. domesticus* on inhibiting the activity of the enzyme, regardless of the method and solvent of extraction. Additionally, in agreement with the current study, the solvent of extraction did not impact on the inhibitory activity. However, the compounds responsible for this inhibitory activity remain unknown [16]. Therefore, further studies would be necessary to clarify the specific compounds contained in the insect extracts that might inhibit the pancreatic lipase. In this sense, our current study contributes to the novel finding that the defatting process does not seem to be a relevant factor on such bioactivity, which suggests that different compounds of either high or low polarity might be involved in the inhibition of the pancreatic lipase.

As summary, these results evidence the potential of extracts obtained from the edible insect *T. molitor* as antioxidants and inhibitors of the pancreatic lipase, a simultaneous multibioactivity that might be favoured by the defatting pre-treatment of the samples and the solvent of extraction. Thus, the most interesting multibioactive extracts of *T. molitor* would be those obtained after a previous defatting process and subsequently extracted with ethanol.

### 3.2. Effect of Defatting on the Yield and Bioactivity of Ethanol Extracts of T. molitor vs. H. illucens

Taking into account the conclusions observed for the extracts in the previous section, a comparative study between the UAE extracts from two different insect species was performed. This was done in order to evaluate if the effects observed after the defatting process would take place regardless of the insect species, as well as to show potential differences between species, regardless of the defatting process. For this assay, two popular edible species, *T. molitor* and *H. illucens*, were compared, and ethanol was selected as solvent of extraction.

**Figure 3:**
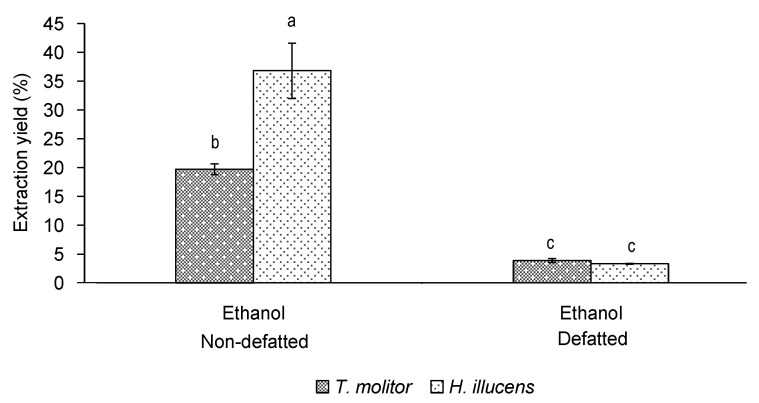
Extraction yield (%, *n* = 2) of non-defatted or defatted *T. molitor* extracts and *H. illucens* extracts obtained by ultrasound-assisted extraction with ethanol. Standard deviations are indicated by error bars. Mean values with different letters are significantly different (*p* ≤ 0.05).

As shown in Figure 3, the higher extraction yield of the non-defatted samples previously observed for *T. molitor* was also confirmed for *H. illucens* ethanol extracts, though significant differences were observed between species. Thus, the extraction yield of non-defatted *H. illucens* was higher than that of *T. molitor* (around 37% and 20%, respectively). This result was probably related to the fact that *H. illucens* was a sample with a higher initial lipid content than *T. molitor* (data not shown), the extraction of such lipid fraction being favoured with ethanol. However, in the case of defatted samples, the extraction yield in both species was the same using ethanol as extraction solvent (Figure 3). Therefore, regardless of the insect species, extracts with similar extraction yields (close to 3%) can be obtained by UAE with ethanol if the defatting of the flours is previously performed.

**Figure 4:**
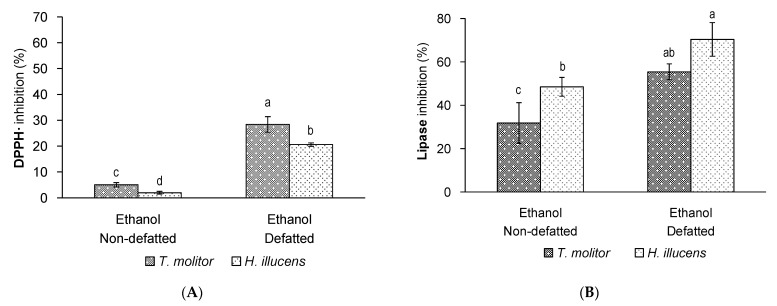
(**A**) DPPH inhibition (%, *n* = 6) and (**B**) pancreatic lipase inhibition (%, *n* = 3) of non-defatted or defatted *T. molitor* extracts and *H. illucens* extracts obtained by ultrasound-assisted extraction with ethanol. Standard deviations are indicated by error bars. Mean values with different letters are significantly different (*p* ≤ 0.05).

Concerning the antioxidant activity, the first result to be remarked in Figure 4A is that ethanol extracts of *H. illucens* showed antioxidant activity, and that the antioxidant mean value was similar to that of *T. molitor* (*p* = 0.245). Additionally, a significant effect of the defatting process was observed (*p* < 0.001). Thus, in general, the antioxidant activity of the defatted samples was higher than that of non-defatted samples, regardless of the insect species (Figure 4A). Nevertheless, the *T. molitor* defatted extract displayed a significantly higher antioxidant activity than that of *H. illucens* (around 28% and 20% of DPPH inhibition, respectively). As already explained, previous studies dealing with antioxidant extracts from edible insects are scarce, and most of them are related to insect flours (mainly as defatted insect flours). Therefore, this study contributes to novel information about the potential of food products from the edible insect for human health. Additionally, further studies elucidating the main compounds responsible for the antioxidant activity of edible insects would be of interest. As we explained above, we previously found a significant correlation between the DPPH inhibition and the total phenolic compounds of *T. molitor* non-defatted extracts [16]. In such experiments, we found that for total phenolic content (TPC) values above 2 g of gallic acid equivalents/100 g of extract, the DPPH inhibition seemed to reach a plateau closer to 90% inhibition. Considering this result, it can be thought that the phenolic content of the assayed extracts in the current study would be lower, given the lower DPPH inhibition values obtained (Figure 4A).

Regarding the inhibitory activity on lipase, both *H. illucens* extracts showed an interesting bioactivity against this enzyme (Figure 4B). In fact, a significantly higher inhibitory activity was observed for *H. illucens* compared to *T. molitor*, regardless of the defatting process (*p* = 0.006). The strongest extract in terms of its inhibitory activity was the defatted one from *H. illucens* (closer to 70% enzymatic inhibition). In fact, considering the defatting factor and regardless of the insect species, a significantly higher inhibitory activity was observed for the defatted samples (*p* < 0.001), in agreement with the same effect observed for the antioxidant activity. These results are very interesting, since we have demonstrated for the first time the inhibitory activity against pancreatic lipase by *H. illucens* extracts. Therefore, these accumulated evidence confirm that different species of edible insects might be potential candidates to produce bioactive extracts that might limit the absorption of dietary lipids by inhibiting the pancreatic lipase. These results also contribute to the understanding of the biological properties of edible insects, since it has been suggested that lipid metabolism can be affected by certain insects species, even though the exact mechanism has not been elucidated. In this regard, antiadipogenic and antiobesity effects have been observed in mice fed aqueous solutions of *T. molitor* and *Allomyrina dichotoma* [12,31]. Additionally, an improvement of the hypercholesterolemia and atherosclerosis in rabbits fed extracts of *Bombyx mori* cocoons has been reported by Ali and Arumugam [32]. However, the specific compounds responsible for the inhibitory activity of insect extracts should be elucidated in further studies, as well as if such compounds are similar or structurally different among the different insect species with demonstrated lipase-inhibitory activity.

In summary, this comparative study between the two common edible insect species consumed in their larvae form confirms that the defatting process of the raw sample is a recommended procedure to obtain multibioactive ethanol extracts from both edible insects. Additionally, *T. molitor* and *H. illucens* might be candidates to produce extracts with simultaneous antioxidant and lipase-inhibitory activities, even though from the antioxidant point of view, the defatted ethanol extract from *T. molitor* would be slightly better; whereas from the lipase-inhibitory point of view, the defatted ethanol extract from *H. illucens* would be slightly preferable.

### 3.3. Effect of Aqueous Ethanol Mixtures on the Yield and Bioactivity of Extracts from Defatted H. illucens

In the general research field of natural extracts, it is frequently found the common use of different aqueous ethanol solutions instead of ethanol for a higher extraction of more polar compounds mainly related to antioxidant and other bioactive properties, as occurs for phenolic compounds [33,34]. Therefore, given the positive results observed for the defatted ethanol extracts of the assayed insects in terms of their bioactivity (Figure 4A,B), we considered it of interest to gain a deeper insight into the effect of different aqueous ethanol solutions on the production of insect extracts. For this assay, *H. illucens* was chosen over *T. molitor* due to its general superior bioactivity in the inhibition of the pancreatic lipase (Figure 4B), which is a very novel bioactivity barely described for insects and worth to be explored in depth.

**Figure 5:**
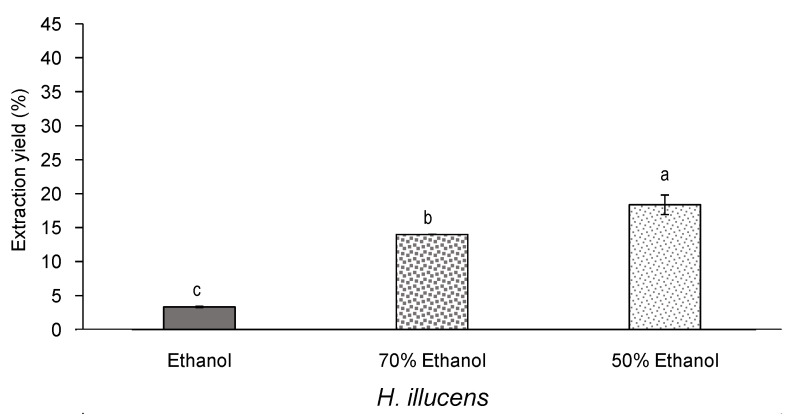
Extraction yield (%, *n* = 2) of defatted *H. illucens* extracts obtained by ultrasound-assisted extraction with ethanol or aqueous ethanol. Standard deviations are indicated by error bars. Mean values with different letters are significantly different (*p* ≤ 0.05).

First, focusing on the extraction yield, a progressive increase was observed along with the polarity of the solvent due to the higher proportion of water mixed with ethanol (Figure 5). Thus, the extraction yield of *H. illucens* varied from around 3% when extracted with 100% ethanol, to around 18% when extracted with 50% aqueous ethanol. This result has been also typically observed for natural extracts from vegetable sources. As example, Metrou-Amir et al. [34] found that the extraction yield varied from around 9% to around 35% when the plant *Matricaria pubescens* was extracted with pure ethanol and aqueous ethanol (50%), respectively.

**Figure 6:**
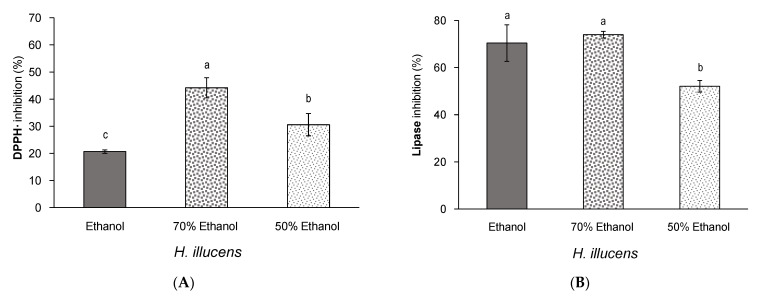
(**A**) DPPH inhibition (%, *n* = 6) and (**B**) pancreatic lipase inhibition (%, *n* = 3) of defatted *H. illucens* extracts obtained by ultrasound-assisted extraction with ethanol or aqueous ethanol. Standard deviations are indicated by error bars. Mean values with different letters are significantly different (*p* ≤ 0.05).

Concerning the DPPH· inhibition, the extraction with aqueous ethanol at both concentrations assayed improved the antioxidant activity of the *H. illucens* extracts compared to 100% ethanol (Figure 6A). In fact, a slight increase in the polarity of the ethanol solution (from 100% ethanol to 70% ethanol) significantly improved the bioactivity of the extracts from around 20% inhibition of the DPPH· radical to nearly 45% inhibition, respectively (Figure 6A).

This would be an interesting result, since it suggests that it would be possible to obtain more effective antioxidant extracts from *H. illucens* together with a lower environmental impact, owing to the use of lower amounts of ethanol. However, a much higher polarity of the ethanol solution (50% ethanol) did not improve the antioxidant activity of the resulting extract compared to the one obtained with 70% ethanol (Figure 6A). Similar effects in terms of the extraction solvent and the antioxidant activity have been observed for extracts from defatted wheat germ, in which the 70% ethanol extract showed the highest DPPH· inhibition over other aqueous ethanol solutions ranging between 0% and 100% ethanol [35]. Therefore, the obtained results suggest that aqueous ethanol solutions with a low aqueous proportion would be preferred over higher aqueous proportions or pure ethanol to obtain antioxidant extracts from *H. illucens*.

Concerning the inhibitory activity against pancreatic lipase by *H. illucens*, it was observed that extracts obtained with both 100% and 70% ethanol were equally effective at inhibiting this enzyme, given the same magnitude of lipase inhibition (around 70%) (Figure 6B). It is also interesting to remark that a superior polarity of the ethanol solution (50% ethanol) did not improve but rather worsened this bioactivity of the extracts. These results suggest that the extracted compounds responsible for this activity are characterised by a low to moderate polarity. Additionally, the result observed for the 70% aqueous ethanol extract would be also of great interest from the sustainability and economical point of view, since it would be possible to produce a higher amount of extract compared to the amount produced with 100% ethanol (14% and 3% extraction yield, respectively), with around seven times less use of ethanol, but as effective as the extract obtained with 100% ethanol.

In summary, aqueous ethanol solutions would be preferred to obtain stronger multibioactive extracts of defatted *H. illucens* compared to ethanol. Solutions of 70% ethanol would also be more adequate than those containing 50% ethanol, since both the antioxidant activity and the inhibition of pancreatic lipase enzyme would be improved simultaneously.

### 3.4. Chemical Composition of Ethanol and Aqueous Ethanol Extracts from Defatted H. illucens

Taking into account the interesting results observed for the ethanol and aqueous ethanol extracts of *H. illucens*, it was considered relevant to characterise some of the main compounds of these extracts by a general GC-MS-FID procedure in order to find differences between the three different *H. illucens* extracts and potential relationships with the biological activities.

The analysis was performed by GC-MS following previous formation of trimethylsilyl derivatives of all those fewer volatile compounds containing hydroxyl or carboxyl functional groups. This procedure allowed to tentatively identify up to 40 compounds (Table 1). Compounds were classified into five groups according to their principal chemical family. Thus, nitrogen compounds, carbohydrates, lipids, organic acids and sterols were identified. As it is clearly shown in Table 1, relevant quantitative and qualitative differences were observed on the chromatographic profile of the three extracts.

The 100% ethanol extract contained a remarkable amount of free amino acids and derivatives (around 7%). Up to 14 different amino acids were identified, although they were mainly represented by proline and its derivative as pyroglutamic acid (L-proline, 5-oxo-), followed by tyrosine, tryptophan, valine and minor amounts of other amino acids. Interestingly, when 70% aqueous ethanol was used for extraction, the total amino acid concentration of the extract almost doubled (around 12%), due to the significant increase of most individual amino acids. This general amino acid enrichment might be explained by the increased polarity of the extraction solvent, allowing the extraction of these more polar compounds. Due to this effect, pyroglutamic acid became the major quantified compound of the 70% extract (5.3%). As far as we know, previous studies describing the presence of this cyclic amino acid derivative in edible insects are scarce. We already described its presence in different non-defatted extracts obtained under different conditions from *A. domesticus* and *T. molitor*, but the chromatographic abundance was in general lower compared to the current extracts of defatted *H. illucens,* except for the aqueous ethanol extract from *T. molitor* obtained by UAE, whose chromatographic abundance was higher (approximately 2 × 10^7^ vs. 3 × 10^7^) [16]. Interestingly, Urbanek et al. [36] also found the presence of this compound in the hygroscopic secretion produced by the secretory setae of terrestrial larvae of the biting midge *Forcipomyia nigra* and successfully demonstrated the antimicrobial activity of pyroglutamic acid on different species of microorganisms. In other natural sources, it has been suggested either as metabolite from microorganisms, or as a natural moisturiser that can be found at high levels in the skin of mammals [37]. Concerning interesting biological properties, an anti-diabetic effect in rats has been related to this compound [37], and the antimicrobial and antiproliferative activity of pyroglutamic acid has been also recently reported [38].

Following amino acids, disaccharides were the second most abundant compounds of the 100% ethanol extract (around 5%), mainly represented by sucrose. As occurred for amino acids, when 70% aqueous ethanol solvent was used for extraction, disaccharides doubled (10.5%) due to the increased polarity of the solvent. Within carbohydrates, the sugar alcohol inositol was also found. According to Vesala et al. [39], these polyols, together with other low molecular weight compounds, as sugars, free amino acids, organic acids and free fatty acids, form an important class of cryoprotectants in insects for cold stress tolerance. Curiously, most of these chemical families, except free fatty acids, were those mainly concentrated in the 70% ethanol extracts obtained from *H. illucens*. Therefore, it can be concluded that the 70% aqueous ethanol extraction of defatted *H. illucens* caused a general enrichment in more polar and hydrosoluble compounds of the extracts, due to the increased polarity of the aqueous mixture.

Concerning the 50% ethanol extract, it was considerably different in terms of the content of amino acids, disaccharides and the rest of the compounds. Only 20 of the total 40 compounds were detected in this extract, and almost the entire extract (98.5%) contained compounds different from those analysable by the used procedure, given that only 1.5% of the extract was quantified, compared to 15% total quantification of the 100% ethanol extract and 27% total quantification of the 70% ethanol extract. Therefore, further studies would be of interest in order to gain a deeper insight into the main composition of all these extracts, and especially into the one obtained with 50% ethanol.

Concerning other minor compounds in the extracts, lipids were also quantified in 100% and 70% ethanol extracts at similar concentrations (1.9% and 1.3%, respectively). The low value of this fraction was expected, due to the previously performed defatted process. This family was mainly represented by free fatty acids under the form of palmitic and oleic acids, together with a minor content of monoglycerides.

Related to lipids, another chemical family worth to be mentioned were sterols. The 100% ethanol extracts contained 0.4% of these compounds, mainly represented by β-sitosterol. Despite the scarce information available on sterols in edible insects, in general, cholesterol used to be the major one described, as it is a typical compound found in most animal sources. However, it is remarkable that *H. illucens* is an atypical animal that tends to accumulate phytosterols from the diet, mainly under the form of β-sitosterol, with lower amounts of cholesterol [40,41,42]. Our obtained extracts successfully demonstrated such expected sterol profile. As an interesting result, 100% ethanol extract also showed the presence of ergosterol. Although its concentration was low, it is worth to remark that this typical fungisterol has not been described in edible insects such as *H. illucens* before, and its potential origin might be related to the diet of the animals. Therefore, taking into account the bioactive implications of phytosterols and ergosterol, mainly as hypocholesterolemic compounds, the obtained enrichment of the 100% ethanol extracts of *H. illucens* would be of interest for this health property. Additionally, since the concentration of phytosterols was reached after a defatting process, this would suggest that the defatting step with hexane was either not fully effective for the complete removal of phytosterols or was not selective. When 70% aqueous ethanol was used for extraction, the phytosterol content of the extracts decreased up to trace values (<0.1%) as expected.

Finally, the 70% ethanol extract contained a minor presence of organic acids (1.3%) that were not found in the 100% ethanol extract. This family mainly consisted of isocitric, gluconic and malic acids. In agreement, we already found the presence of organic acids in non-defatted extracts of *T. molitor* and *A. domesticus* [16].

As summary, it seemed that the extracts from *H. illucens* obtained by 70% ethanol led to products richer in a major diversity of different chemical compounds, mainly of more polar or semipolar nature.

In order to detect potential relationships between the chemical composition of the extracts and their previously demonstrated biological activities, Pearson’s correlation tests were performed between each quantified compound and their inhibitory activity of pancreatic lipase or DPPH radical. Only one significant positive correlation was found, which was between the amino acid proline and the inhibitory activity of pancreatic lipase (*r* = 0.999; *p* = 0.022). This was an intriguing result to explain since the inhibitory activity of this enzyme by free amino acids is not a typical effect. Nevertheless, Pradhan et al. [43] already described a significant correlation between the inhibition of pancreatic lipase and this same amino acid from the methanol extracts of fruit pulps of *Mangifera indica*. Additionally, these authors also found a positive effect of alanine and aspartic acid, but these amino acids were not found in the *H. illucens* extracts. It should be remarked that despite the significant correlation observed in our current study, it should not be discarded that other compounds which were not in the extracts might be also involved in the biological activities of lipase and DPPH inhibition, as well as potential synergies likely taking place between the myriad of compounds contained in the extracts.

## 4. Conclusions

The present study shows the potential of extracts obtained from the edible insects *T. molitor* and *H. illucens* as antioxidants and inhibitors of the pancreatic lipase, a simultaneous multibioactivity of interest for human health. This multibioactivity might be favoured by the defatting pre-treatment of the samples and the use of specific solvents of extraction. Thus, in general, the defatting process favours the antioxidant activity and inhibition of pancreatic lipase, regardless of the solvent used for extraction and the insect species. Concerning the solvent of extraction, methanol seems to be the most interesting one from the antioxidant point of view, but ethanol and, specially, aqueous ethanol at 70%, would be preferred for a better antioxidant and lipase-inhibitory simultaneous multibioactivity. In terms of the two assayed species, in general, defatted ethanol extracts from *T. molitor* might be preferred for antioxidant activities, whereas those from *H. illlucens* would be preferred for inhibition of pancreatic lipase. The chemical characterisation of defatted aqueous ethanol extracts from *H. illucens* shows a great diversity of compounds, including amino acids, disaccharides, lipids, organic acids and sterols, which are specially concentrated in the 70% ethanol extract. Due to such complexity, further studies are still necessary to clearly establish a relationship between the chemical composition and the bioactivities of these innovative natural extracts from edible insects.

This study will potentiate the development of novel insect-based food products for human health, since it shows that the production of effective insect extracts as bioactive ingredients is possible, especially from previously defatted samples and by using green and sustainable solvents of extraction. Nevertheless, this study can be considered almost a first step, taking into account the great diversity of edible insect species that can be explored and the large number of variable factors and processing conditions that can be still studied in the emerging general field of natural extracts obtained from edible insects.

## Figures and Tables

**Table 1:** GC-MS characterisation and quantitation (g/100 g extract) of extracts from *H. illucens*.

Rt	Compound	Ethanol		70% Ethanol		50% Ethanol	
		Area ^1^	g/100 g	Area ^1^	g/100 g	Area ^1^	g/100 g
	NITROGEN COMPOUNDS		6.98		12.31		0.27
8.452	1-Aminocyclopropane-1-carboxylic acid	247,215	0.060 ± 0.000 ^a^	441,580	0.109 ± 0.018 ^a^	n.d.	n.d.
8.941	L-Valine	1,318,699	0.318 ± 0.008 ^a^	1,356,182	0.335 ± 0.020 ^a^	n.d.	n.d.
9.704	L-Proline	5,984,738	1.443 ± 0.034 ^a^	7,246,143	1.790 ± 0.158 ^a^	125,239	0.030 ± 0.013 ^b^
10.265	Serine	361,032	0.087 ± 0.001 ^a^	1,130,996	0.279 ± 0.025 ^b^	17,938	0.004 ± 0.003 ^c^
10.503	Threonine	499,614	0.120 ± 0.000 ^a^	867,255	0.214 ± 0.012 ^b^	16,597	0.004 ± 0.003 ^c^
11.016	L-Homoserine	220,070	0.053 ± 0.006 ^a^	308,851	0.076 ± 0.009 ^a^	n.d.	n.d.
11.664	Pyroglutamic acid	11,601,355	2.797 ± 0.050 ^a^	21,341,912	5.271 ± 0.464 ^b^	584,034	0.140 ± 0.055 ^c^
11.824	L-Norvaline	661,970	0.160 ± 0.004 ^a^	1,042,372	0.257 ± 0.020 ^b^	n.d.	n.d.
12.317	Ornithine	98,721	0.024 ± 0.000 ^a^	587,180	0.145 ± 0.021 ^b^	n.d.	n.d.
12.358	Glutamine	125,877	0.030 ± 0.000 ^a^	1,476,601	0.365 ± 0.031 ^b^	n.d.	n.d.
12.411	Phenylalanine	180,696	0.044 ± 0.001 ^a^	192,340	0.047 ± 0.000 ^b^	n.d.	n.d.
12.748	Asparagine	n.d.	n.d.	304,685	0.075 ± 0.016	n.d.	n.d.
13.117	L-Lysine	79,662	0.019 ± 0.002 ^a^	701,187	0.173 ± 0.014 ^b^	n.d.	n.d.
14.641	L-Tyrosine	5,214,872	1.258 ± 0.077 ^a^	10,036,215	2.478 ± 0.159 ^b^	368,497	0.089 ± 0.081 ^c^
14.510	L-Histidine	547,412	0.132 ± 0.001 ^a^	1,222,452	0.302 ± 0.047 ^b^	n.d.	n.d.
16.346	L-Tryptophan	1,819,631	0.439 ± 0.039 ^a^	1,583,493	0.392 ± 0.082 ^a^	n.d.	n.d.
	CARBOHYDRATES		5.77		11.72		0.43
15.344	Inositol	1,181,332	0.638 ± 0.002 ^a^	2,148,282	1.190 ± 0.155 ^b^	n.d.	n.d.
18.328	Disaccharide n.i.	n.d.	n.d.	5,546,267	1.020 ± 0.045	171,438	0.031 ± 0.014
18.603	D-Turanose	2,770,474	0.498 ± 0.016 ^a^	15,594,801	2.881 ± 0.972 ^b^	460,240	0.083 ± 0.041 ^c^
19.067	Disaccharide n.i.	n.d.	n.d.	1,615,927	0.297 ± 0.012 ^a^	93,107	0.017 ± 0.001 ^b^
19.193	Disaccharide n.i.	4,100,860	0.737 ± 0.030 ^a^	12,577,917	2.318 ± 0.376 ^b^	994,620	0.178 ± 0.044 ^c^
19.279	Sucrose	21,682,632	3.895 ± 0.120 ^a^	21,782,315	4.015 ± 0.799 ^a^	708,107	0.130 ± 0.056 ^b^
	LIPIDS		1.89		1.29		0.76
9.794	Butanedioic acid	171,695	0.023 ± 0.001 ^a^	659,873	0.090 ± 0.008 ^b^	25,606	0.003 ± 0.001 ^c^
12.518	Dodecanoic acid	1,200,162	0.159 ± 0.005 ^a^	373,726	0.051 ± 0.003 ^b^	138,970	0.018 ± 0.003 ^c^
13.909	Tetradecanoic acid	1,287,237	0.171 ± 0.006 ^a^	573,640	0.078 ± 0.010 ^b^	178,096	0.024 ± 0.004 ^c^
15.337	Hexadecanoic acid	3,113,733	0.413 ± 0.030 ^a^	1,554,915	0.211 ± 0.016 ^b^	863,370	0.114 ± 0.041 ^b^
16.351	Linoleic acid	737,090	0.098 ± 0.001 ^a^	n.d.	n.d.	87,952	0.012 ± 0.002 ^b^
16.499	Oleic acid	2,712,012	0.360 ± 0.011 ^a^	1,541,780	0.209 ± 0.035 ^b^	610,728	0.081 ± 0.003 ^c^
18.211	2-Monopalmitin	n.d.	n.d.	n.d.	n.d.	262,082	0.056 ± 0.000
18.380	1-Monopalmitin	2,849,658	0.579 ± 0.014 ^a^	3,141,554	0.651 ± 0.119 ^a^	1,933,553	0.400 ± 0.021 ^a^
19.141	2-Monostearin	n.d.	n.d.	n.d.	n.d.	243,208	0.052 ± 0.002
19.044	Monoglyceride n.i.	335,835	0.072 ± 0.003	n.d.	n.d.	n.d.	n.d.
20.238	Hexacosanoic acid	94,318	0.013 ± 0.002	n.d.	n.d.	n.d.	n.d.
	ACIDS		0.00		1.31		0.04
11.337	Malic acid	n.d.	n.d.	313,005	0.140 ± 0.022	n.d.	n.d.
13.855	Isocitric acid	n.d.	n.d.	1,661,187	0.743 ± 0.141 ^a^	101,811	0.044 ± 0.013 ^b^
15.139	Gluconic acid	n.d.	n.d.	951,216	0.425 ± 0.049	n.d.	n.d.
	STEROLS		0.40		0.09		-
21.381	Ergosterol	136,147	0.091 ± 0.006	n.d.	n.d.	n.d.	n.d.
21.487	Campesterol	121,618	0.081 ± 0.006	n.d.	n.d.	n.d.	n.d.
21.687	Stigmasterol	48,518	0.032 ± 0.002 ^a^	17,156	0.012 ± 0.004 ^b^	n.d.	n.d.
22.036	β-Sitosterol	296,052	0.197 ± 0.015 ^a^	10,9096	0.075 ± 0.015 ^b^	n.d.	n.d.

^1^ Mean value (*n* = 2); different superscript letters within a row mean statistical differences between the extracts (*p* ≤ 0.05); n.d. = not detected, n.i. = not identified.
